# Iron Chelator Induces Apoptosis in Osteosarcoma Cells by Disrupting Intracellular Iron Homeostasis and Activating the MAPK Pathway

**DOI:** 10.3390/ijms22137168

**Published:** 2021-07-02

**Authors:** Yanru Xue, Gejing Zhang, Shoujie Zhou, Shenghang Wang, Huanhuan Lv, Liangfu Zhou, Peng Shang

**Affiliations:** 1 School of Life Science, Northwestern Polytechnical University, Xi’an 710072, China; xueyanru@mail.nwpu.edu.cn (Y.X.); zgj@mail.nwpu.edu.cn (G.Z.); zhoushaojie613@mail.nwpu.edu.cn (S.Z.); wangshenghang@mail.nwpu.edu.cn (S.W.); lvhh2017@nwpu.edu.cn (H.L.); zlf19900919@mail.nwpu.edu.cn (L.Z.); 2 Research & Development Institute of Northwestern Polytechnical University in Shenzhen, Shenzhen 518057, China; 3 Key Laboratory for Space Bioscience and Biotechnology, Institute of Special Environment Biophysics, Northwestern Polytechnical University, Xi’an 710072, China

**Keywords:** iron chelators, iron metabolism, ROS, osteosarcoma, MAPK signaling pathway, apoptosis

## Abstract

Osteosarcoma is a common malignant bone tumor in clinical orthopedics. Iron chelators have inhibitory effects on many cancers, but their effects and mechanisms in osteosarcoma are still uncertain. Our in vitro results show that deferoxamine (DFO) and deferasirox (DFX), two iron chelators, significantly inhibited the proliferation of osteosarcoma cells (MG-63, MNNG/HOS and K7M2). The viability of osteosarcoma cells was decreased by DFO and DFX in a concentration-dependent manner. DFO and DFX generated reactive oxygen species (ROS), altered iron metabolism and triggered apoptosis in osteosarcoma cells. Iron chelator-induced apoptosis was due to the activation of the MAPK signaling pathway, with increased phosphorylation levels of JNK, p38 and ERK, and ROS generation; in this process, the expression of C-caspase-3 and C-PARP increased. In an orthotopic osteosarcoma transplantation model, iron chelators (20 mg/kg every day, Ip, for 14 days) significantly inhibited the growth of the tumor. Immunohistochemical analysis showed that iron metabolism was altered, apoptosis was promoted, and malignant proliferation was reduced with iron chelators in the tumor tissues. In conclusion, we observed that iron chelators induced apoptosis in osteosarcoma by activating the ROS-related MAPK signaling pathway. Because iron is crucial for cell proliferation, iron chelators may provide a novel therapeutic strategy for osteosarcoma.

## 1. Introduction

Osteosarcoma is a primary mesenchymal tumor histologically characterized by malignant cells that produce osteoid. Osteosarcoma commonly occurs in the long bones of the extremities near the metaphyseal growth plates. The age distribution of osteosarcoma is bimodal, with the first peak in adolescence and the second peak in adults over 65 years. With the introduction of combination chemotherapy in the 1970s, the overall 5-year survival rate of osteosarcoma increased from 10–20% to 60–70% [1]. However, the survival rate among metastatic patients has remained 20–30% in the past two decades [2]. Consequently, it is essential to explore new and effective treatment strategies.

Iron is an essential element and involved in important physiological processes necessary for life [3,4]. Abnormal iron metabolism is a characteristic of most cancer cells, including breast, lung and prostate cancers. The abnormally high “iron content” in cells also affects therapeutic efficacy and cancer prognosis [5,6]. Cancer cells generally exhibit abnormal iron metabolism and increased iron demand to maintain their malignant proliferation. Iron intake, efflux, storage and regulatory pathways are all disordered in cancer, which indicates that iron metabolism is key to tumor cell survival [7]. These findings suggest the need for a new cancer therapy strategy that targets iron metabolism.

Iron plays an important role in oxidative stress, and targeting iron has received interest as a potential cancer treatment [8,9]. Iron chelators were originally developed to primarily treat diseases related to iron overload [10,11,12,13,14]; however, in recent years, their therapeutic potential in treating cancer has emerged. Studies have shown that iron chelators have an antiproliferative effect in myeloid leukemia cells and lymphoma cells [15,16]. DFX was able to reduce tumor burden in two mouse models of lung and esophagus cancers [17,18]. Furthermore, when combined with chemotherapeutic drugs, DFX greatly enhanced the effect of esophageal chemotherapy. Iron chelators can form redox-active metal complexes, which can cause oxidative stress by generating reactive oxygen species, destroy key intracellular targets and cause cell apoptosis [19]. Moreover, iron chelators induced ROS production in gastric cancer cells, resulting in apoptosis via the endoplasmic reticulum (ER) stress pathway [20]. To date, a few studies have demonstrated the efficacy of iron deprivation in osteosarcoma models [21]. However, the level of evidence for the effectiveness of iron chelators as anti-tumor adjuvants in osteosarcoma treatment appears to be insufficient to alter clinical practice. Therefore, the effect of iron chelators on osteosarcoma is worth studying.

ROS are closely related to tumor cell death [22]. ROS promote tumor development by inducing DNA mutation and genomic instability or, as signaling molecules, by accelerating tumor cell proliferation, survival and metastasis. The “free” or “catalyzed” form of iron mediates the production of reactive oxygen species and causes oxidative stress through the Fenton reaction. Iron-induced oxidative stress leads to two possible outcomes: (1) redox regulation failure, leading to lipid peroxidation and oxidative DNA and protein damage; (2) redox regulation, which activates various protective mechanisms to reduce iron and oxidative stress. A growing number of studies have reported a correlation between increased iron storage and increased cancer [23]. However, excessive ROS enhance oxidative stress, causing damage to DNA, proteins and lipids and triggering cell apoptosis or necrosis [24]. Hence, increasing the level of ROS in tumor cells with chemotherapeutic drugs has been applied to the clinical treatment of cancer. The unstable iron pool in the cell directly catalyzes the generation of ROS through the Fenton reaction [25]. Cells contain a large number of ROS sources, including iron-dependent ROS activation. Iron is a key component of several ROS-producing enzymes, such as NADPH oxidase (NOXs), lipoxygenase (LOXs), cytochrome P450 (CYP) and mitochondrial electron transport chain subunits. Excess intracellular iron can be stored in ferritin, where it is isolated and cannot participate in ROS-generating reactions. Ferritin includes two subunits, ferritin heavy chain (FTH) and ferritin light chain (FTL). The destruction of ferritin leads to an iron-dependent increase in ROS and cell death, including apoptosis, necrosis and ferroptosis [22,26]. Apoptosis is programmed cell death, starting and completing in an ordered manner by activating and/or synthesizing the gene product required for synthesizing cells [27]. The MAPK, Bcl-2 and cysteine-dependent aspartate-specific protease (Caspase) families are closely related to the apoptosis process [28]. Studies have indicated that the MAPK family, including c-Jun N-terminal kinase (C-JNK), p38 mitogen-activated protein kinase (p38 MAPK) and p-ERK1/2, play important roles in the regulation of oxidative stress-induced apoptosis [29,30]. 

In this study, we aimed to delve into the molecular mechanisms involved in the anticancer effects of iron chelators in osteosarcoma cells for a comprehensive understanding of this process. Our results demonstrate that, in iron chelator-treated osteosarcoma cells, iron metabolism altered, ROS increased, and the MAPK signaling pathway was activated, triggering apoptosis.

## 2. Results

### 2.1. Iron Chelators DFO and DFX Inhibited Viability of Osteosarcoma Cells and Proliferation In Vitro 

**Figure 1:**
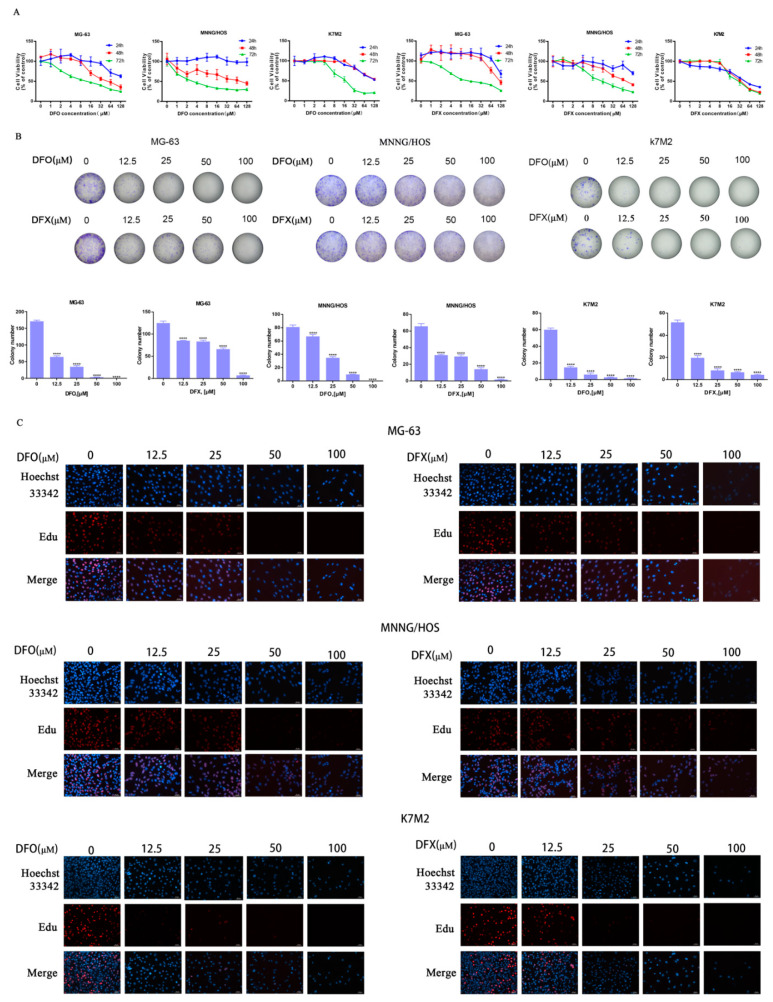
Iron chelators inhibited cell viability and proliferation of osteosarcoma cells. (**A**) Viability of MG-63, MNNG/HOS and K7M2 cells treated with a series of concentrations of DFO and DFX for 24 h, 48 h or 72 h. The data are presented as mean ± SD (n = 5). (**B**) Colony formation assay of MG-63, MNNG/HOS and k7M2 cells treated with DFO and DFX. The data are presented as mean ± SD (n = 3). (**C**) EdU staining assay of MG-63, MNNG/HOS and K7M2 cells treated with DFO and DFX for 48 h. **** *p* < 0.0001 versus control group.

To investigate the effects of iron chelators on the viability of osteosarcoma cells, we used the CCK-8 assay kit. MG-63, MNNG/HOS and K7M2 cells were treated with increasing concentrations of DFO and DFX (0, 12.5, 25, 50, 100 μM). The CCK-8 analysis results in Figure 1A show that DFO and DFX reduced MG-63, MNNG/HOS and K7M2 cell viability in a dose- and time-dependent manner. Colony numbers of MG-63, MNNG/HOS and K7M2 cells decreased with 24 h DFO or DFX treatment (Figure 1B). Similarly, as the concentration of iron chelators increased, colony formation was significantly inhibited. DFO and DFX at 50 mM completely abolished colony formation in MG-63, MNNG/HOS and K7M2 (Figure 1B). These results suggest that DFO or DFX can significantly inhibit the colony-forming efficiency of MG3-63, MNNG/HOS and K7M2 cells. Using the EdU incorporation assay, we further investigated the ability of iron chelators to decrease the proliferation of MG-63, MNNG/HOS and K7M2 cells. We observed a significant dose-dependent decrease in EdU-positive MG-63, MNNG/HOS and K7M2 cells treated with DFO or DFX compared to the control (Figure 1C). Taken together, these results demonstrate that DFO and DFX may inhibit cell viability and proliferation in MG-63, MNNG/HOS and K7M2 osteosarcoma cells.

### 2.2. Iron Chelators Induced Cell-Cycle Arrest in Osteosarcoma Cells

**Figure 2:**
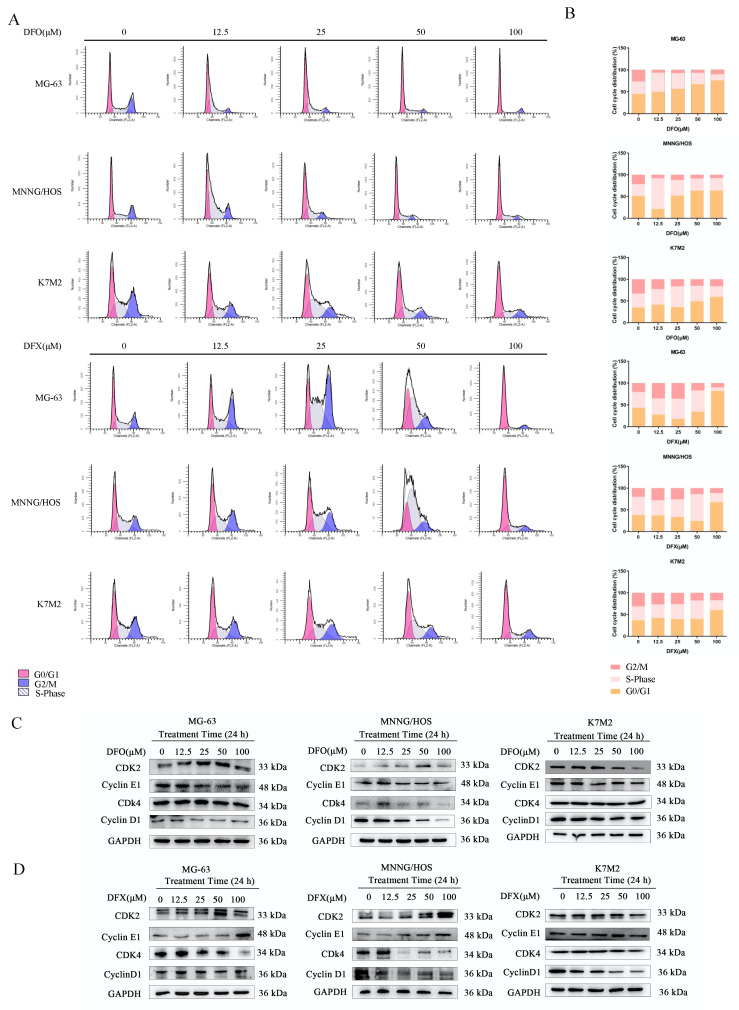
DFO induced G0/G1 cell-cycle arrest, and DFX induced S cell-cycle arrest in osteosarcoma cells. (**A**) The cell-cycle distribution of MG-63, MNNG/HOS and K7M2 cells after treatment with the indicated concentrations of DFO and DFX for 24 h, visualized by PI staining. (**B**) Quantitative analysis of cell-cycle distribution in (**A**). (**C**,**D**) Protein expression levels of CDK2, cyclin E1, CDK4 and cyclin D1 in MG-63, MNNG/HOS and K7M2 osteosarcoma cells treated with the indicated concentrations of DFO and DFX for 24 h.

An abnormal cell cycle can activate the apoptotic pathway. Therefore, we monitored the cell cycle in osteosarcoma cells treated with DFO and DFX for 24 h to investigate the effects of the iron chelators on the cell cycle (Figure 2A,B). DFO treatment increased the G0/G1-phase fraction of MG-63, MNNG/HOS and K7M2 osteosarcoma cells. However, DFX induced a rise in the S-fraction of cells. Cell-cycle progression is regulated by cyclin-dependent protein kinases (CDKs) and their regulatory subunits, cyclins [31]. To further determine the mechanism of DFO and DFX effects on the cell cycle, we measured the expression of cell-cycle-related regulatory proteins after treatment with the iron chelators. Western blots showed that 24 h DFO and DFX treatment markedly decreased the expression of cyclin D1 and cyclin-dependent kinase 4 (CDK4) in osteosarcoma cells, but CDK4 expression not significantly decreased in K7M2 cell. CDK2 expression increased at low DFO concentrations and decreased at high DFO concentrations, and cyclin E levels were reduced. The expression of cyclin E1 was suppressed by DFO but not significantly decreased by DFX; an increase in cyclin E protein levels was observed after DFX treatment (Figure 2C,D). Taken together, these results demonstrate that DFO and DFX may cause cell apoptosis by triggering the dysregulation of the cell cycle.

### 2.3. Iron Chelators Altered Iron Metabolism in Osteosarcoma Cells

**Figure 3:**
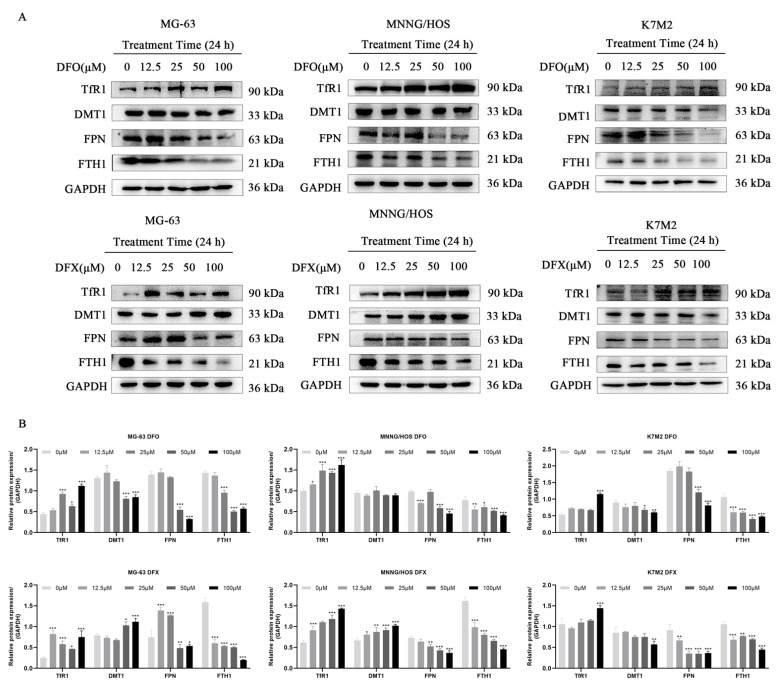
Iron chelators altered iron metabolism in osteosarcoma cells. (**A**) Protein expression levels of TfR1, DMT1, FTH1 and FPN in MG-63, MNNG/HOS and K7M2 cells treated with the indicated concentrations of DFO and DFX for 24 h. (**B**) Quantitative analysis of TfR1, DMT1, FPN and FTH1 in MG-63, MNNG/HOS and K7M2 cells treated with the indicated concentrations of DFO and DFX for 24 h. * *p* < 0.05, ** *p* < 0.01 and *** *p* < 0.001 versus control group.

Iron is an essential nutrient element with a variety of biological functions, including oxygen binding, electron transfer and acting as a catalyst for hundreds of enzymes [32]. Therefore, we analyzed the effects of iron chelators on iron metabolism in MG-63, MNNG/HOS and K7M2 osteosarcoma cells (Figure 3). After DFO treatment, the protein expression of DMT1 not significantly changed in MNNG/HOS osteosarcoma cell, TfR1 was upregulated, and FPN, FTH1 and DMT1 were downregulated in osteosarcoma cells. Similarly, DFX treatment led to a dose-dependent increase in the expression of TfR1 and the downregulation of FPN and FTH1 in MG-63, MNNG/HOS and K7M2 osteosarcoma cells. However, DMT1 expression increased in MG-63 and MNNG/HOS and decreased in K7M2 after DFX treatment (Figure 3A,B). All of these data indicate that DFO and DFX alter iron metabolism in osteosarcoma cells.

### 2.4. Iron Chelators Induced Oxidative Stress in Osteosarcoma Cells

**Figure 4:**
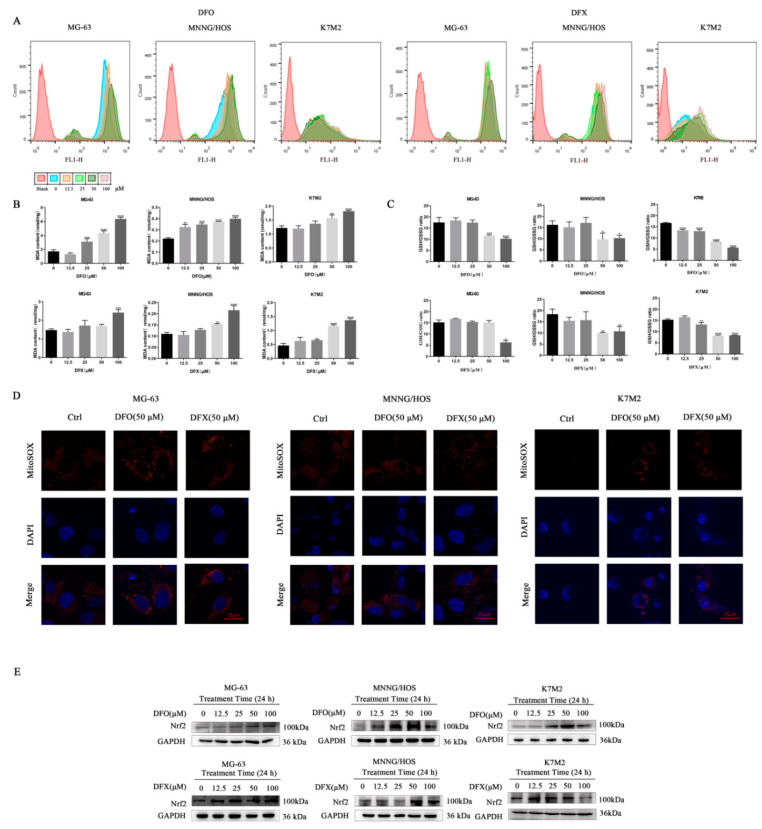
Iron chelators induced oxidative stress in osteosarcoma cells. (**A**) ROS levels in MG-63, MNNG/HOS and K7M2 cells treated with the indicated concentrations of DFO and DFX for 24 h, detected by using a DCFH-DA sensor. (**B**) MDA level in MG-63, MNNG/HOS and K7M2 cells treated with the indicated concentrations of DFO and DFX for 24 h. (**C**) GSH/GSSG ratio in MG-63, MNNG/HOS and K7M2 cells treated with the indicated concentrations of DFO and DFX for 24 h. (**D**) Mitochondrial superoxide production in MG-63, MNNG/HOS and K7M2 cells treated with the indicated concentrations of DFO and DFX for 24 h, observed by a confocal laser scanning microscope. (**E**) Protein expression levels of Nrf2 in MG-63, MNNG/HOS and K7M2 cells treated with the indicated concentrations of DFO and DFX for 24 h. * *p* < 0.05, ** *p* < 0.01, *** *p* < 0.001 and **** *p* < 0.0001 versus control group.

The intracellular labile iron pool (LIP) directly catalyzes the generation of ROS through the Fenton reaction [33]. To evaluate the redox state in osteosarcoma cells treated with iron chelators, we tested cellular ROS levels using a DCFH-DA sensor. The results indicate that 24 h DFO treatment notably increased cellular ROS levels in MG-63 and MNNG/HOS osteosarcoma cells, and DFX treatment notably increased cellular ROS levels in MG-63, MNNG/HOS and K7M2 osteosarcoma cells (Figure 4A). To verify whether ROS induced by iron chelators exerted oxidative stress in osteosarcoma cells, we measured the level of MDA, an end product that is generated by lipid peroxidation. MDA generation was significantly enhanced by DFO and DFX treatment in MG-63, MNNG/HOS and K7M2 osteosarcoma cells (Figure 4B). To clarify whether the mechanism by which DFO and DFX induced oxidative stress in MG-63, MNNG/HOS and K7M2 osteosarcoma cells originated from the depletion of GSH, we further measured the ratio of reduced GSH to GSSG in osteosarcoma cells treated with DFO for 24 h. Increased concentrations of DFO and DFX dramatically reduced the GSH/GSSG ratio in MG-63, MNNG/HOS and K7M2 osteosarcoma cells (Figure 4C). Additionally, superoxide in the mitochondria of osteosarcoma cells was detected by a fluorescent mitochondrial superoxide marker, MitoSOXTM Red M36008. Increased MitoSOX fluorescence was found in MG-63, MNNG/HOS and K7M2 osteosarcoma cells after DFO and DFX (50 µM) treatment for 24 h (Figure 4D). NF-E2-related factor 2 (Nrf2) is a critical transcription factor regulating oxidative stress [34]. Therefore, we examined Nrf2 expression in osteosarcoma cells. Western blots showed that DFO and DFX treatment upregulated the expression of Nrf2 (Figure 4E). Collectively, these results demonstrate that iron chelators can induce oxidative stress in osteosarcoma cells.

### 2.5. Iron Chelators Induced Apoptosis in Osteosarcoma Cells

**Figure 5:**
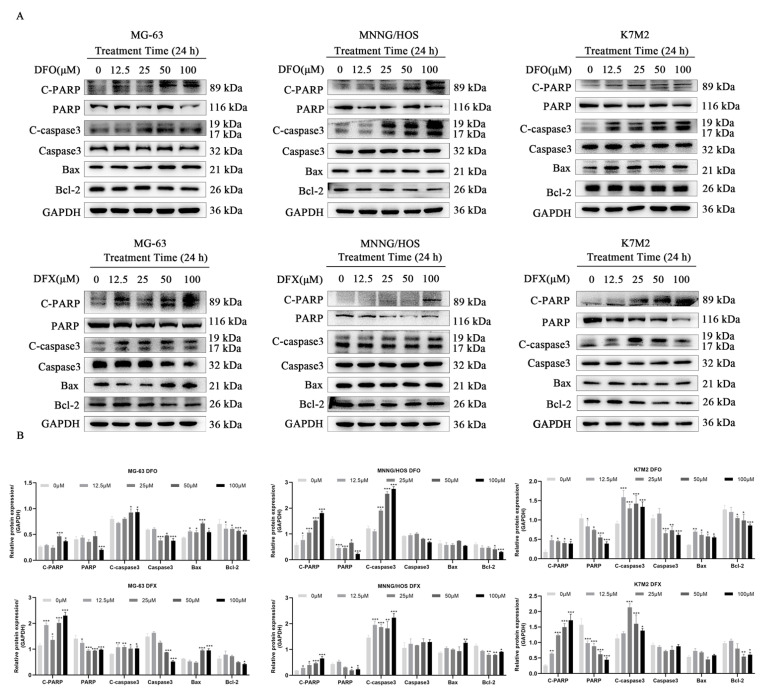
Iron chelators induced apoptosis in osteosarcoma cells. (**A**) Protein expression levels of Caspase-3, C-caspase-3, Bcl2, Bax, and C-PARP in MG-63, MNNG/HOS and K7M2 cells treated with the indicated concentrations of DFO and DFX for 24 h. (**B**) Quantitative analysis of C-PARP, PARP, C-caspase-3, caspase-3, Bax and Bcl2 in MG-63, MNNG/HOS and K7M2 cells treated with the indicated concentrations of DFO and DFX for 24 h (n = 3). * *p* < 0.05, ** *p* < 0.01 and *** *p* < 0.001 versus control group.

Several studies have reported that iron chelators can significantly affect intracellular and extracellular iron levels and cause tumor cell apoptosis [15,17,18,20,35]. To verify the mechanism underlying iron chelator-induced anti-proliferation effects in osteosarcoma cells, we used Western blots to evaluate the apoptotic profiles of MG-63, MNNG/HOS and K7M2 cells. The Western blot results show that the iron chelators DFO and DFX promoted caspase-3 activation and significantly increased the levels of C-PARP and Bax and decreased the levels of Bcl-2 and PARP in osteosarcoma cells (Figure 5A,B). These results indicate that osteosarcoma cells undergo apoptosis after iron chelator treatment.

### 2.6. DFO and DFX Induced Apoptosis by Activating the Ros-Mediated Mapk Pathway in Osteosarcoma Cells

**Figure 6:**
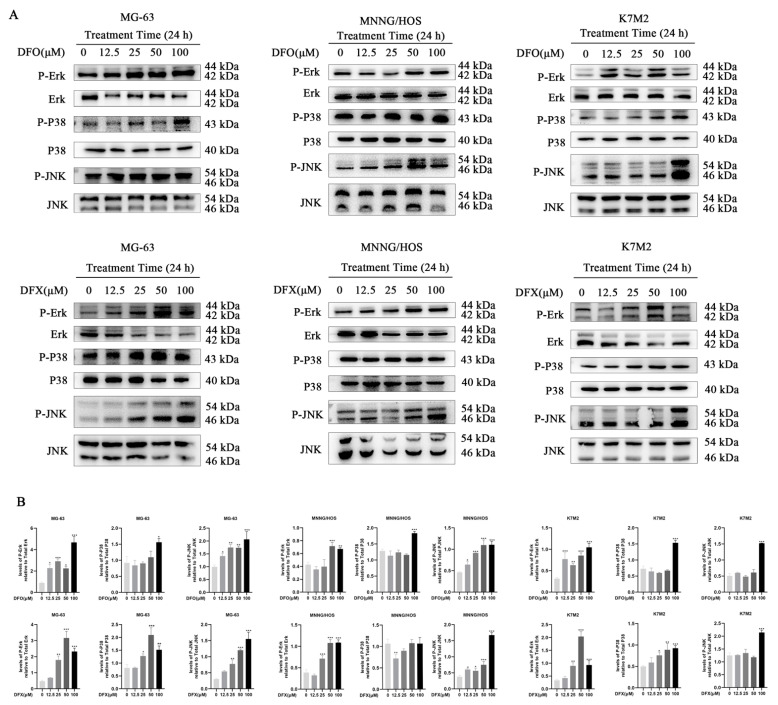
Iron chelators activated the MAPK signaling pathway in osteosarcoma cells. (**A**) Phosphorylation levels of p-Erk, p38 and JNK in MG-63, MNNG/HOS and K7M2 cells treated with the indicated concentrations of DFO and DFX for 24 h. (**B**) Quantitative analysis of P-Erk, Erk, P-P38, P38, P-JNK and JNK in MG-63, MNNG/HOS and K7M2 cells treated with the indicated concentrations of DFO and DFX for 24 h (n = 3). * *p* < 0.05, ** *p* < 0.01 and *** *p* < 0.001 versus control group.

MAPKs, including P38, JNK and ERK1/2, have been implicated in the regulation of the ER stress response and apoptosis. Studies have reported the involvement of the MAPK pathway during iron chelator-mediated apoptotic cell death [36]. Therefore, we assessed whether MAPKs were activated in iron-chelator-treated osteosarcoma cells. We found that iron chelators activated the MAPK signaling pathway in osteosarcoma cells by phosphorylating JNK, P38 and ERK1/2. Iron chelators activated JNK, P38 and ERK1/2 as concentrations increased (Figure 6A,B). These results indicate that the MAPK signaling pathway was activated in iron-chelator-treated osteosarcoma cells. To further evaluate the activation of the MAPK pathway in vivo, we analyzed the expression of p-p38, p-JNK and p-ERK1/2 in osteosarcoma tissues with or without iron chelator treatment. These results demonstrate that the MAPK signaling pathway was activated in iron-chelator-treated osteosarcoma.

### 2.7. Iron Chelators Exerted Anti-Tumor Effects in Mice with Osteosarcoma Tumor Allografts

**Figure 7:**
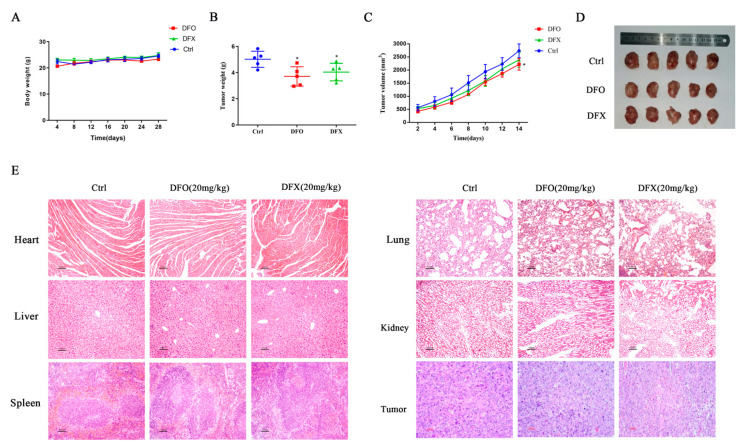
Iron chelators inhibited the growth of osteosarcoma in vivo. K7M2 cells were injected in situ into the bone marrow cavity of the right tibia of male BALB/c mice. Nine days after the establishment of the osteosarcoma xenotransplantation mouse model, the mice were randomly divided into 3 groups and were given normal saline and 20 mg/kg DFO or DFX once a day for two consecutive weeks. (**A**) Body weight changes in all groups. (**B**) The weights of the 3 groups of tumor tissues. (**C**) Volume changes in the 3 groups of tumor tissues. The data were calculated by the following formula: volume = length × width^2^ × 1/2. (**D**) The 3 groups of tumor tissues. (**E**) H&E staining analysis of heart, liver, spleen, lung, kidney and tumor tissues (200× and 400×). All data are expressed as mean ± SD (n = 5). * *p* < 0.05 versus control group.

**Figure 8:**
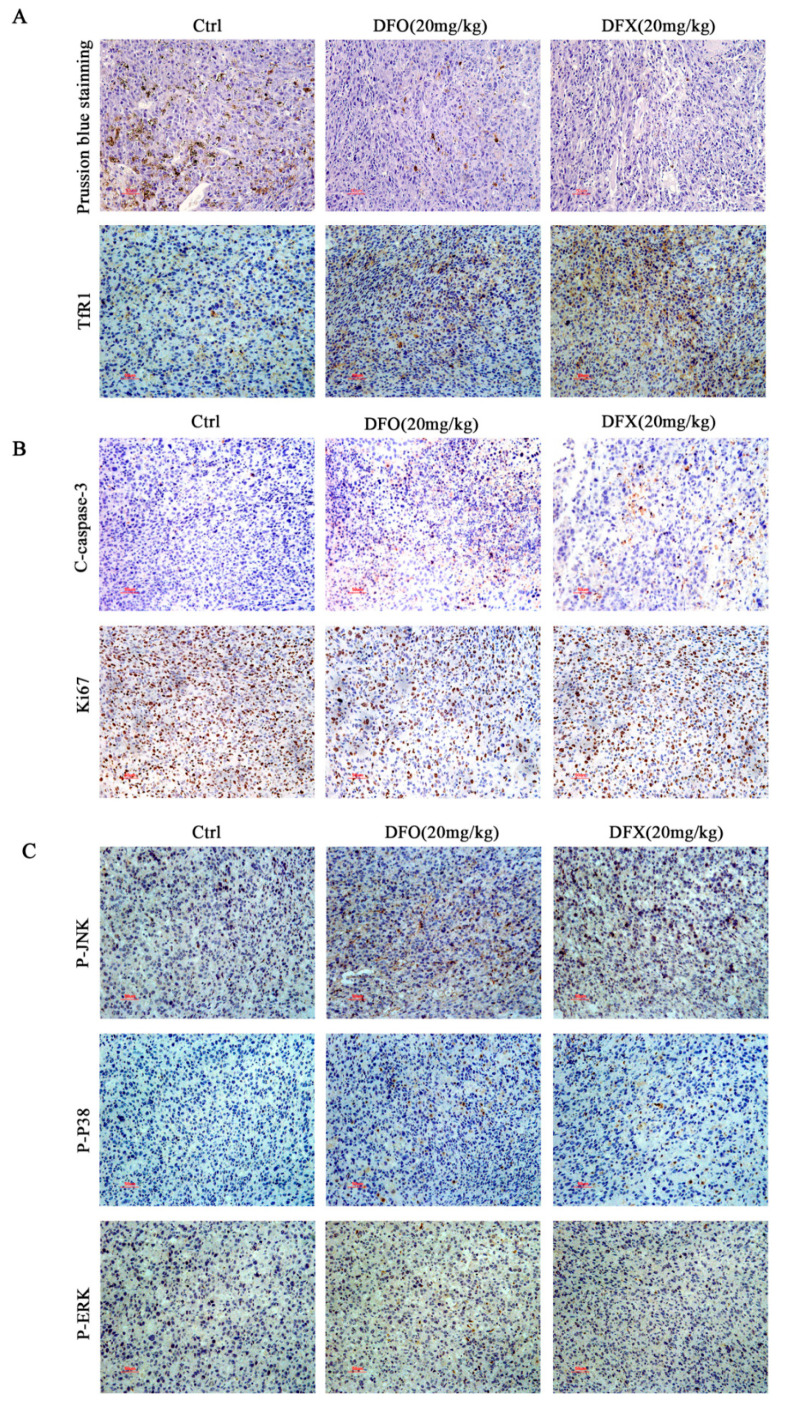
Effect of iron chelators on iron metabolism and MAPK pathway in tumor tissue. (**A**) Prussian blue staining analysis of tumor tissue and expression of TfR1 in tumor tissues. (**B**) C-caspase-3 and Ki67 immunohistochemical analysis. (C) P-JNK, P-P38 and P-ERK1/2 immunohistochemical analysis.

To investigate the antiproliferative activity of iron chelators in vivo, we examined whether DFO and DFX could inhibit the growth of K7M2 in an osteosarcoma allograft model. We found no significant differences in weight between the groups (Figure 7A). At a concentration of 20 mg/kg, DFO or DFX significantly inhibited tumor growth after 14 days of drug treatment compared to the control (Figure 7B–D). Furthermore, H&E staining of tumor sections showed a high degree of cancerous necrosis in the control group. H&E staining of the heart, liver, spleen, lung and kidney showed no signs of toxicity compared to the control group (Figure 7E). Prussian blue staining of the tumor tissues showed that the iron content was significantly reduced after the iron chelator treatment. Immunohistochemical analysis showed that after DFO and DFX drug treatment, TfR1 expression in tumor tissue significantly increased compared to the control (Figure 8A). These data indicate that DFO and DFX altered iron metabolism in tumor tissues. Ki67 staining showed decreased proliferation of osteosarcoma cells, and the levels of C-caspase-3 in tumor tissue increased after DFO and DFX drug treatment (Figure 8B). Immunohistochemical analysis detected significant activation of P-P38, P-JNK and P-ERK1/2 expression in tumor tissue with DFO or DFX treatment compared to the control (Figure 8C). These results are consistent with the previous in vitro results. Together, the data show that iron chelators exert a notable tumor suppressor effect in osteosarcoma mice with low systemic toxicity by activating MAPK cell death signaling pathways.

## 3. Discussion

Iron is an essential nutrient that contributes to cellular functions such as cell proliferation and growth. Studies have shown that elevated levels of iron in the human body can increase the risk of cancer and death [37]. In addition, iron can accelerate tumor initiation and tumor growth [5]. Iron-chelating agents can effectively remove iron content in cells, so they have received widespread attention as potential tumor treatment [38]. The iron chelators DFO and DFX have been widely used in clinical settings as treatments for patients with iron-overload-related diseases. Numerous studies have shown that iron chelators have anti-tumor potential in different cancers, including human breast adenocarcinoma, human colon cancer, hepatocellular carcinoma, human malignant lymphoma and leukemia, and clinical studies have previously demonstrated excellent safety profiles, which makes DFO and DFX effective therapeutic agents for cancer treatment [16,39,40,41]. Osteosarcoma is a common malignant tumor, but current treatment methods are limited and mainly rely on surgery, radiotherapy and chemotherapy. Previous studies have shown that iron chelators induced apoptosis and autophagy in cancer cells via caspase activation and JNK/NFκB pathway activation. However, the mechanisms in osteosarcoma are not fully understood. In this study, we found that the redox homeostasis of MG-63 and MNNG/HOS human osteosarcoma cells and K7M2 murine osteosarcoma cells responded to iron chelators. The iron chelators DFO and DFX inhibited cell proliferation by increasing cellular ROS levels, and they effectively induced apoptosis by disrupting intracellular iron homeostasis and activating the MAPK pathway in MG-63 and MNNG/HOS human osteosarcoma cells and K7M2 murine osteosarcoma cells.

The side effects of iron chelator treatment were studied, and no significant alterations in the functions of various organs, including heart, liver, spleen, lung and kidney, were detected in treatment groups compared with the control group. Several iron-chelating agents have been approved as drugs by the FDA. DFX is generally well tolerated in humans [42,43]. In terms of their side effects, no significant changes in the functions of various organs were found in our study. These results are consistent with previous studies [17,35], demonstrating the safety of DFO and DFX as monotherapies in tumor treatments. Generally speaking, our findings indicate that DFO and DFX are well tolerated in mice.

ROS-driven caspase-dependent apoptosis was the major mechanism of cell death. DFO and DFX have induced apoptosis in melanoma and hepatoma cells, leukemias and neuroblastomas [44,45]. In our study, 24 h DFO and DFX treatment notably increased cellular ROS levels in osteosarcoma cells in a concentration-dependent manner. However, the present study had some limitations: we did not establish how DFO and DFX could cause iron deficiency and increase mitochondrial ROS. Previously, it was reported that DFO-induced iron-deficient conditions and increased mitochondrial iron levels in triple-negative MDA-MB-231 breast cancer cells could generate large amounts of ROS [46]. Hence, we speculate that iron chelators may increase the level of mitochondrial iron, which will cause osteosarcoma cells to produce a large amount of ROS, eventually increasing the level of mitochondrial oxidative stress and ultimately inducing cell apoptosis. We evaluated the expression of caspase-3, PARP, Bcl-2 and Bax by Western blotting to investigate apoptosis in MG-63 and MNNG/HOS human osteosarcoma cell lines and K7M2 cells after 24 h incubation with DFO or DFX. The results show that DFO and DFX promoted caspase-3 activation, significantly increased the levels of C-PARP and Bax and decreased the levels of Bcl-2 and PARP. These results indicate that osteosarcoma cells undergo apoptosis after iron chelator treatment.

DFO and DFX are known to induce cell death [20]. Previous studies have indicated that cyclin D1 overexpression occurred early in the oral tumorigenesis process and was significantly associated with advanced tumor stages [47]. Iron chelators induced S-phase cell-cycle arrest [21]. Fukuchi cultured ML-1 and Raji cells with 30–100 μM DFO for 24–48 h and found that the cells were blocked in the G0/G1 phase [48], while DFO-treated neuroblastoma (NB) cells were in the cell cycle G1 phase, which is the early stage of DNA synthesis [49]. Renton’s research demonstrated that, according to the DFO concentration and the length of exposure time, glioma cells were blocked in the G1/S or G2/M stage [50]. Our results show that DFO treatment significantly inhibited cell growth and caused G0/G1-phase cell-cycle arrest, and DFX treatment significantly inhibited cell growth and caused S-phase cell-cycle arrest. Cyclin D1, a key cell-cycle control protein, was decreased by the iron chelators, which indicates that they induced cell-cycle arrest. Although the expression of cyclin E1 was suppressed by DFO, DFX did not significantly suppress its expression. The differing expression of cyclin E protein may reflect dysregulation of the cell cycle in MG-63, MNNG/HOS and K7M2 osteosarcoma cells treated by the indicated concentrations of DFO and DFX for 24 h. The reason for the increased expression of CDK2 at low DFO and DFX concentrations and the decrease at higher concentrations remains unclear. It can be speculated that, at low DFO concentrations, a compensatory increase in expression may occur in response to the cell-cycle arrest. Further detailed studies are required to elucidate the precise molecular mechanisms involved.

Previous studies on the effect of iron chelators on body iron or tumor iron storage have produced inconsistent results. Several studies demonstrated that iron chelator treatment has an effect on systemic iron and tumor iron storage. In our study, after DFO treatment, TfR1 expression increased significantly, and FTH1, FPN and DMT1 expression decreased; however, DMT1 expression increased after DFX treatment in human osteosarcoma cells in vitro. The analyses also revealed that iron chelator treatment disturbed the redox balance in MG-63, MNNG/HOS and K7M2 cells by decreasing GSH levels and increasing ROS levels, which also indicates that iron deprivation promotes ROS-dependent apoptosis mechanisms in vitro. Taken together, these results suggest that the apoptosis mechanism of DFO- and DFX-induced iron deficiency in osteosarcoma is complex, and further studies are required to clarify the precise molecular mechanisms involved.

## 4. Materials and Methods

### 4.1. Cell Culture and Chemicals

MG-63 and MNNG/HOS human osteosarcoma cell lines and the K7M2 murine osteosarcoma cell line were obtained from the Cell Bank of Type Culture Collection of Chinese Academy of Sciences. The cells were cultured in a 5% CO_2_ incubator at 37 °C in Dulbecco’s modified Eagle’s medium (DMEM, Gibco, Gaithersburg, USA) supplemented with 10% fetal calf serum and 1% penicillin/streptomycin antibiotics. The iron chelators DFO and DFX were procured from MedChemExpress (Monmouth Junction, NJ, USA).

### 4.2. Cell Viability Assay

MG-63, MNNG/HOS and K7M2 cells were seeded at 2.5 × 10^4^ cells/mL in 96-well plates and cultured overnight. Then, cells were treated with DFO or DFX (0, 12.5, 25, 50, 100 μM) for 24, 48 or 72 h. DFO was dissolved in PBS, and DFX was dissolved in DMSO. The cell viability assay was performed with the Cell Counting Kit 8 assay according to the manufacturer’s protocols. The plates were read by a Synergy HT multimode microplate reader (BioTek, Winooski, VT, USA) at a wavelength of 450 nm.

### 4.3. Colony Formation Assay

A colony formation assay was used to assess the anti-growth efficacy of DFO and DFX in osteosarcoma cells. The osteosarcoma cells were cultured in a 6-well plate at 5 × 10^2^ cells/mL and then treated with different concentrations (0, 12.5, 25, 50, 100 μM) of DFO or DFX for 24 h. The medium was replaced with fresh medium every three days for a continuous cultivation period of ten days. The colonies were fixed with 4% paraformaldehyde for 10 min and stained with 0.5% crystal violet. A stereo microscope was used to observe colony formation.

### 4.4. Cell Cycle Analysis

The cell cycle was detected using the Cell Cycle and Apoptosis Analysis Kit (Beyotime, C1052) by flow cytometry. MG-63, MNNG/HOS and K7M2 cells were seeded in a 6-well plate at 1 × 10^5^ cells/mL and adhered overnight. The cells were treated with DFO or DFX (0, 12.5, 25, 50, 100 μM) for 24 h. Cells were rinsed with pre-cooled 1× PBS and then trypsinized and collected. The cells were recentrifuged at 1000× *g* for 3 min, washed once with cold 1× PBS, resuspended in 1 mL of pre-chilled 70% ethanol and stored at 4 °C for 12 h. After washing the cells with cold PBS, 500 μL of PI staining solution was added to each sample and incubated for 30 min at 37 °C in the dark. The samples were tested with a FACSCalibur flow cytometer (BD Biosciences, San Jose, CA, USA), and ModFit software was used to calculate the percentage of cells in each stage of the cell cycle.

### 4.5. Measurement of Cytosolic ROS

Intracellular ROS levels were detected with the ROS Assay Kit (Beyotime Biotechnology, China) according to the manufacturer’s protocols. Osteosarcoma cells were seeded in a 6-well plate with a seeding density of 1 × 10^5^ cells/mL. After overnight adherence and subsequent treatment with DFO or DFX (0, 12.5, 25, 50, 100 μM) for 24 h, MG-63, MNNG/HOS and K7M2 cells were collected and incubated with a DCFH-DA sensor for 30 min at 37 °C while protected from light. The stained cells were washed twice with PBS and analyzed by flow cytometry (BD Biosciences, San Jose, CA, USA).

### 4.6. Assessment of Mitochondrial Superoxide Production

MitoSOX™ Red (M36008, Invitrogen, Australia) was used to evaluate mitochondrially derived superoxide in osteosarcoma cells. After DFO or DFX (0, 12.5, 25, 50, 100 μM) treatment for 24 h, MG-63, MNNG/HOS and K7M2 cells were incubated in DMEM with MitoSOX™ Red M36008 (5 μM) and DAPI at 37 °C for 40 min. The stained cells were washed twice with PBS. Stained cells were observed with a confocal laser scanning microscope (TCS, SP5, Leica Microsystems, Wetzlar, Germany).

### 4.7. Measurement of Malondialdehyde

Malondialdehyde (MDA) levels were measured by using a lipid peroxidation MDA assay kit (Beyotime Biotechnology, China). After DFO or DFX (0, 12.5, 25, 50, 100 μM) treatment for 24 h, MG-63, MNNG/HOS and K7M2 cells were washed with 1× PBS, lysed with RIPA lysis buffer and centrifuged at 12,000× *g* for 10 min to obtain the supernatant. The lysed cell preparation step was performed at 4 °C. After the sample preparation was completed, the protein concentration was determined using the BCA protein assay. Subsequently, the absorbance was measured at 532 nm using a microplate reader. The calculated protein content per unit weight represents the MDA content in the original sample.

### 4.8. Measurement of GSH/GSSG

The level of GSH/GSSH was detected by using the GSH/GSSG assay kit (Beyotime, Shanghai, China). After DFO or DFX (0, 12.5, 25, 50, 100 μM) treatment for 24 h, MG-63, MNNG/HOS and K7M2 cells were washed with 1× PBS and recentrifuged at 1000× *g* for 3 min, and the supernatant was aspirated. The cells were lysed by two freeze–thaw cycles in liquid nitrogen and a 37 °C water bath. The detection principle of this kit is that GSH reacts with the chromogenic substrate DTNB to produce yellow TNB. The amount of total cell GSH can be calculated by detecting the absorbance at 412 nm with a microplate reader. The levels of GSH and GSSG were detected in the osteosarcoma cells according to the operating steps of the kit. The molar concentrations of GSH and GSSG were calculated according to the standard curve, and the GSH and GSSG contents were determined as the protein content per unit weight.

### 4.9. Western Blot Analysis

MG-63, MNNG/HOS and K7M2 cells were seeded at 1.5 × 10^5^ cells/mL in 6-well plates and treated with DFO or DFX with concentration gradients (0, 12.5, 25, 50, 100 μM). After DFO or DFX treatment for 24 h, protein was extracted from cells using radioimmunoprecipitation assay (RIPA; Beyotime, Shanghai, China) lysate. Immunoreactive bands were visualized by enhanced chemiluminescence (ECL) Detection System (T5200, Tanon). Antibodies against FTH1 (75972), Bcl2 (18858), Bax (182733) and Nrf2 (62352) were purchased from Abcam (Cambridge, MA, USA). Antibodies against TfR1 (13-6800), FPN (PA5-22993) and DMT1 (PA5-35136) were purchased from Thermo Fisher Scientific (Waltham, MA, USA). Antibodies against ERK (4348S), JNK (9252S), p-JNK (9251S), p38 (11451S) and p-p38 (4092S) were purchased from Cell Signaling Technology (Danvers, MA, USA). Antibodies against GAPDH (AF0006), Caspase-3 (AC030), C-caspase-3 (AC033), C-PARP (AF1567), CDK2 (AF1603), CDK4 (AF2515), cyclin D1 (AF1183) and cyclin E1 (AF2491) were purchased from Beyotime Biotechnology (Shanghai, China). Antibody against P-ERK (AP0472) was purchased from ABclonal Technology (Boston, MA, USA).

### 4.10. Orthotopic Transplantation Tumors of k7M2 in Balb/C Mice

Five-week-old male BALB/c mice were purchased from Beijing Vital River Laboratory Animal Technology Co. Ltd. One week later, K7M2 cells (1 × 10^6^ /100 μL) were injected into the bone marrow cavity of the tibia. Animals were cared for in accordance with institution guidelines. The animal study was reviewed and approved by the medical and experimental animal ethics committee of Northwestern Polytechnic University. After 10 days, the tumors were established, and the mice with orthotopic tumor volumes (≥100 mm^3^) were randomized into 3 groups: Ctrl group, DFO group and DFX group (I.P.), with 0.9% normal saline used in the control group and 20 mg/kg DFO or 20 mg/kg DFX administered in the treated groups. The growth of xenografts was measured by using vernier calipers at 2-day intervals. Tumor volume was calculated by the equation (volume = (length × width × (width/2)). Mice were euthanized and sacrificed after two weeks.

### 4.11. Histological Analysis

After the mice were euthanized, the heart, liver, spleen, lung, kidney and tumor of the mice were obtained and fixed in 4% paraformaldehyde for 48 h. Then, the tissues were embedded in paraffin, and 5 μm paraffin sections were obtained via a semiautomated rotary microtome. Hematoxylin and eosin (H&E) staining was performed on the sections. First, the tissue sections were deparaffinized and then treated with 100% (I, II), 90%, 80% and 70% alcohol for 5 min and tap water for 5 min × 3. Hematoxylin staining for 5 min was followed by tap water flushing. Then, for differentiation, sections were treated with 5% acetic acid for 1 min and rinsed with tap water, and acetic acid was added dropwise with a pipette. Eosin staining was followed by rinsing with tap water, dehydrating with 70%, 80%, 90% and 100% alcohol for 10 s each, rinsing with xylene for 1 min and applying neutral gum as the seal.

### 4.12. Immunohistochemistry Analysis

The tumor was fixed in 4% paraformaldehyde-buffered saline and embedded in paraffin for immunohistochemistry. Diluted primary antibody solutions of C-caspase-3, ki67, P-JNK, P-P38, P-ERK and TfR1 were dripped onto paraffin sections. The sections were placed in a wet box and incubated at 4 °C overnight. The slides were placed in PBS (pH 7.4) and washed 3 times on a decolorizing shaker for 5 min per wash. After the sections were slightly dried, the tumor tissues were covered with a secondary antibody (HRP marker) against the corresponding species of the primary antibody and incubated for 50 min at room temperature. After the slices were slightly dried, freshly prepared 3,3′-diaminobenzidine tetrahydrochloride (DAB) was added dropwise, and color development was monitored under a microscope. The positive color was brownish yellow, and the reaction was terminated by rinsing with tap water. After a tap water rinse, the slides were counter-stained with hematoxylin, dehydrated and mounted.

### 4.13. Statistical Analysis

Statistical analyses were performed using GraphPad Prism 8 Software (version 8, GraphPad Software, Inc., La Jolla, CA, USA). All data are expressed as means ± standard deviation (SD). The significance of differences between different experimental groups was determined by using Student’s t-test or one-way ANOVA with Fisher’s LSD multiple comparisons test. * p < 0.05, ** p < 0.01 and *** p < 0.001 vs. the indicated control group were considered significant.

## 5. Conclusions

**Figure 9:**
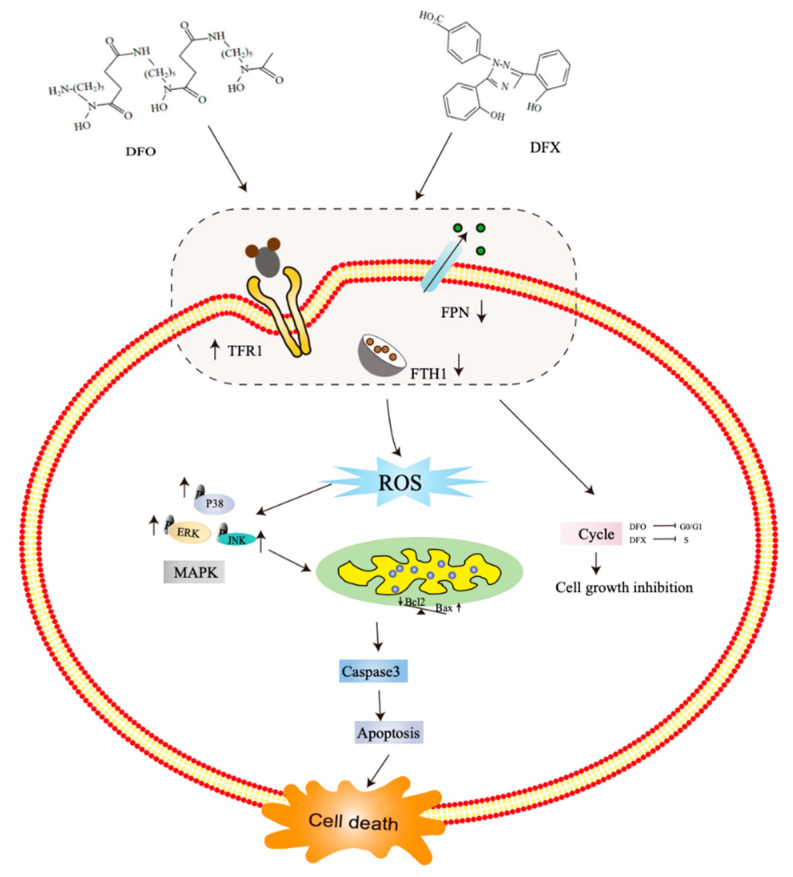
A schematic diagram of the effect of iron chelators on osteosarcoma cells. DFO and DFX altered iron metabolism, released ROS, the activation of the MAPK pathway; DFO induced G0/G1 cell-cycle arrest, DFX induced S cell-cycle arrest, and both iron chelators triggered apoptosis in osteosarcoma cells. These processes induce osteosarcoma cell death.

In summary, iron chelators demonstrated a potent anti-growth effect on osteosarcoma cells in vitro, and DFO and DFX were further shown to inhibit osteosarcoma tumor growth in a xenograft animal model in vivo. DFO and DFX targeted iron metabolism by activating the ROS-related MAPK signaling pathway; DFO induced G0/G1 cell-cycle arrest, DFX induced S cell-cycle arrest, and both iron chelators triggered apoptosis in osteosarcoma cells (Figure 9). Our research results indicate that iron deprivation has potential as a new strategy for osteosarcoma cancer treatment. Targeting iron metabolic pathways may provide new tools for cancer prognosis and therapy.

## Data Availability

The data that support the findings of this study are available from the corresponding author on reasonable request.
